# UV-B Pre-treatment Alters Phenolics Response to *Monilinia fructicola* Infection in a Structure-Dependent Way in Peach Skin

**DOI:** 10.3389/fpls.2018.01598

**Published:** 2018-11-06

**Authors:** Marco Santin, Susanne Neugart, Antonella Castagna, Martina Barilari, Sabrina Sarrocco, Giovanni Vannacci, Monika Schreiner, Annamaria Ranieri

**Affiliations:** ^1^Department of Agriculture, Food and Environment, University of Pisa, Pisa, Italy; ^2^Department of Biological Sciences, Loyola University, New Orleans, LA, United States; ^3^Leibniz Institute of Vegetable and Ornamental Crops (IGZ), Großbeeren, Germany; ^4^Interdepartmental Research Center Nutrafood “Nutraceuticals and Food for Health”, University of Pisa, Pisa, Italy

**Keywords:** flavonol glycosides, ultraviolet radiation, fruit, *Prunus persica*, post-harvest, brown rot

## Abstract

Phenolic compounds represent a large class of secondary metabolites, involved in multiple functions not only in plant life cycle, but also in fruit during post-harvest. phenolics play a key role in the response to biotic and abiotic stresses, thus their accumulation is regulated by the presence of environmental stimuli. The present work aimed to investigate how different pre-UV-B-exposures can modulate the phenolic response of peach fruit infected with *Monilinia fructicola*. Through HPLC-DAD-MS^n^, several procyanidins, phenolic acids, flavonols, and anthocyanins were detected. Both UV-B radiation and fungal infection were able to stimulate the accumulation of phenolics, dependent on the chemical structure. Regarding UV-B exposure, inoculated with sterile water, 3 h of UV-B radiation highest concentration of phenolics was found, especially flavonols and cyanidin-3-glucoside far from the wound. However, wounding decreased the phenolics in the region nearby. When peaches were pre-treated with 1 h of UV-B radiation, the fungus had an additive effect in phenolic accumulation far from the infection, while it had a subtractive effect with 3 h of UV-B radiation, especially for flavonols. Canonical discriminant analysis and Pearson correlation revealed that all phenolic compounds, except procyanidin dimer, were highly regulated by UV-B radiation, with particularly strong correlation for quercetin and kaempferol glycosides, while phenolics correlated with the fungus infection were quercetin-3-galactoside, quercetin-3-glucoside, kaempferol-3-galactoside and isorhamnetin-3-glucoside. Modulation of pathogen-induced phenolics also far from inoculation site might suggest a migration of signaling molecules from the infected area to healthy tissues.

## Introduction

During their lifespan, fruit have to deal with several biotic (e.g., pathogen infections, herbivore attacks) and abiotic (e.g., water deficiency, UV-B radiation, high/low temperature) stresses. In order to tolerate such environmental adverse conditions, plants have developed fine-tuned responses through the synthesis and accumulation of many phytochemicals (Schreiner and Huyskens-Keil, 2006). Among these, phenolic compounds represent one of the most representative class of secondary metabolites, widespread in inflorescences, leaves and fruit (Ehlenfeldt and Prior, 2001; Elfalleh, 2012; Chung et al., 2016; Senica et al., 2017). Phenolics fulfill fundamental functions during plant life cycle, e.g., as signaling molecules, phytoalexins, pigments, antioxidants, and flavor-contributors (Ali Ghasemzadeh, 2011). Among phenolics, flavonoids, and phenolic acids play the main role in defensive mechanisms against microbial and fungal attacks (Rauha et al., 2000; Puupponen-Pimia et al., 2001; Rodríguez Vaquero et al., 2007; Tocci et al., 2018). Cushnie and Lamb (2005) reported that flavonoids act as antimicrobials especially by inhibiting the nucleic acid synthesis, the cytoplasmic membrane function and the energy metabolism. Modulation of phenolic compounds is induced not only by biotic, but also abiotic factors, e.g., UV-B radiation (Liu et al., 2011; Castagna et al., 2013; Scattino et al., 2014; Santin et al., 2018). UV-B radiation activates a specific transduction pathway, which leads to the upregulation of genes involved in phenylpropanoid metabolism, altering the phenolic concentration in both vegatative and generative plant organs (Brown et al., 2005; Favory et al., 2009; Scattino et al., 2014). Previous studies revealed that UV-B positively affects phenolic concentration and profile in many different fruit, such as peach (Scattino et al., 2014; Santin et al., 2018), apple (Lancaster et al., 2000; Fiskaa et al., 2007; Falguera et al., 2011) tomato (Castagna et al., 2014) and lemon (Interdonato et al., 2011). Moreover, recent evidences have reported a phenolic class/compound-dependent response toward UV-B radiation in peach skin (Scattino et al., 2014; Santin et al., 2018). In this sense, Santin et al. (2018) found an increase in anthocyanins, flavones and dihydroflavonols according to the UV-B dose given (1.39 or 8.33 kJ m^−2^), after 36 h of storage. In different peach cultivars and with a 36 h UV-B irradiation, Scattino et al. (2014) showed a different trend of accumulation between hydroxycinnamic acids and flavonols. A positive role of UV-B radiation has been also observed not only as stimulator of antioxidant compounds, but also in extending the shelf-life of post-harvest fruit by lowering the softening process (Scattino et al., 2015). However, effects of UV-B radiation can be very different depending on the UV-B dose given, since a long and intense UV-B exposure might induce non-specific stress responses to the plant, while a mild and short UV-B radiation triggers specific adaptation responses, such as the activation of genes specifically involved in UV-B acclimation (Favory et al., 2009; Jenkins, 2017). However, evidences in literature show that when two stressors are combined, the effects are not simply the sum of both the effects of the two stressors individually (Rizhsky et al., 2004; Mittler, 2006). Indeed, the simultaneous presence of different modifications of environmental conditions might activate different signaling pathways, often with contrasting and complex effects (Asselbergh et al., 2008). Several studies investigated the effect of combination of two or more abiotic stressors on plant phenolics, e.g., heavy metals with high/low temperatures in wheat (Öncel et al., 2000), drought and UV-B exposure in lettuce (Rajabbeigi et al., 2013), UV-C radiation and heat treatment on *Botrytis cinerea* and *Rhizopus stolonifer* growth in strawberry fruit (Pan et al., 2004). However, literature on combined effects of both biotic and abiotic factors on plant phenolics is scarce. Combination of water stress and infection with plant-parasitic nematode in tomato resulted in altering the response of some secondary metabolites among flavonoids and carotenoids, behaving differently from when the two stresses were applied singularly (Atkinson et al., 2011). Studying post-harvest fruit behavior toward combined biotic and abiotic factor is crucial because it reflects better the complex environmental reality that crops has to face daily, with multiple stressors simultaneously. In wheat, it was found that higher mean temperatures recorded over 6 years resulted in a higher susceptibility to many several kinds of infection, such as viral, fungal, and bacterial (Sharma et al., 2007). Similarly, an increased susceptibility toward fungal pathogens was observed in sorghum, common bean and date palm following drought stress (Diourte et al., 1995; McElrone et al., 2001; Suleman et al., 2001; Mayek-Pérez et al., 2002). Abiotic factors might play also a positive role in enhancing the plant defense toward pathogen and pest attacks. It was found that resistance toward *B. cynerea* is increased by water scarcity in tomato fruit (Achuo et al., 2006), and high salinity condition enhanced the resistance toward powdery mildew in barley (Wiese et al., 2004). However, to date very few data are available about the likely positive role of a mild UV-B pre-treatment on counteracting a fungal infection on peach fruit.

One of the most aggressive pathogen for stone fruits is *Monilinia fructicola*, a fungus responsible for the brown rot disease in pre- and post-harvest peach fruit (Guidarelli et al., 2014; Spadoni et al., 2014). Since phenolic compounds play a key role in plant-pathogen interaction (Rodríguez Vaquero et al., 2007; Tocci et al., 2018), and considering the positive effect of UV-B radiation in stimulating phenolic secondary metabolism, this work aimed to investigate whether several different pre-UV-B treatments enhanced the phenolic response induced by the infection by *M. fructicola* in peach fruit.

## Materials and methods

### Plant material and UV-B treatments

Organic peaches (*Prunus persica* cv. Royal Majestic, melting phenotype) were purchased from a local supermarket and brought to the laboratory of the Department of Agriculture, Food and Environment (DAFE), University of Pisa, Pisa (Italy). All peaches were accurately checked and only undamaged ones with homogeneous dimension were used. Fruit were randomly divided into five groups of ten peaches each. UV-B treatments were performed in proper UV-B chambers equipped with three UV-B lamps per chamber (Philips Ultraviolet-B Narrowband, TL 20W/01—RS, Koninklijke Philips Electronics, Eindhoven, The Netherlands; irradiance 1.36 W m^−2^). A constant temperature of 24°C was set inside the chambers. Five separate UV-B treatments were performed: 0 h (control, UVB-0), 1 h (UVB-1), 3 h (UVB-3), 6 h (UVB-6), and 12 h (UVB-12). The chambers for both control and UV-B irradiated groups were equipped also with photosynthetically active radiation (PAR) lamps, which provided white light for all the duration of the UV-B treatments. After the UV-B irradiation, peaches were kept under PAR up to 24 h (e.g., peaches treated for 1 h UV-B+PAR were kept for 23 h more under PAR). The experiment was conducted three times, with five individual fruit each time.

### *M. fructicola* inoculation

*M. fructicola* 10757, kindly given by Marta Mari (University of Bologna, Italy) was used in this study. The fungus was deposited at the fungal collection of the Department of Agricultural Science, Food and Environment (University of Pisa) on Potato Dextrose Agar (PDA, Difco, USA) agar slants under mineral oil at 4°C and, when needed, grown on PDA plates at 24°C under a 12 h photoperiod per day until sporulation occurred. One-week-old cultures were accurately rinsed with sterile water to obtain the conidia suspension, and conidia concentration was adjusted to 1 × 10^5^ conidia per ml.

Peaches were wounded (~1 cm long, ~1 cm deep each wound) with a sterile scalpel and inoculated with 20 μL of *M. fructicola* conidial suspension. Two wounds were made on the UV-B-exposed side of the fruit. For each UV-B treatment, control peaches were inoculated with 20 μL sterile H_2_O. Fruits were incubated at 24°C in plastic bags for 24 h, to maintain a high level of humidity until the drop of conidia suspension was absorbed within the wound. After, bags were removed and peaches were left in the incubator for 2 days more. When the necrotic area was clearly visible on inoculated wounds, peach skin from the UV-B-exposed side of the fruit, both inoculated and not inoculated and far and near the necrotic area/wound, was sampled, dipped in liquid nitrogen and lyophilized until further analyses. The ring portion of the skin (~1 cm wide) just around the necrotic area has been considered as the region “near” *M. fructicola* infected area; a similar ring portion around the not inoculated wound was considered the region “near” to the wound. A circular portion of the skin (~ 1 cm radius) with margin 3 cm far from the necrotic area margin (or from the uninoculated wounds) was sampled as the “far” region.

### Extraction and HPLC-DAD-MS^n^ identification of phenolics

Phenolic compounds were extracted according to Schmidt et al. (2010) with some modification. Lyophilized, ground material (0.02 g) was extracted with 600 μl of 60% aqueous methanol for 40 min at 20°C. The extract was centrifuged at 4,500 rpm for 10 min at the same temperature, and the supernatant was collected in a reaction tube. This process was repeated twice with 300 μl of 60% aqueous methanol for 20 min and 10 min, respectively; the three corresponding supernatants were combined. The extract was subsequently evaporated until dryness and it was then suspended in 200 μl of 10% aqueous methanol. The extract was centrifuged at 3,000 rpm for 5 min at 20°C through a Corning® Costar® Spin-X® plastic centrifuge tube filter (Sigma Aldrich Chemical Co., St. Louis, MO, USA) for the HPLC analysis. Each extraction was carried out in duplicate.

Flavonoid composition (including hydroxycinnamic acid derivatives and glycosides of flavonoids) and concentrations were determined from the filtrate using a series 1100 HPLC (Agilent Technologies, Waldbronn, Germany) equipped with a degasser, binary pump, autosampler, column oven, and photodiode array detector. An Ascentis® Express F5 column (150 mm × 4.6 mm, 5 μm, Supelco) was used to separate the compounds at a temperature of 25°C. Eluent A was 0.5% acetic acid, and eluent B was 100% acetonitrile. The gradient used for eluent B was 5–12% (0–3 min), 12–25% (3–46 min), 25–90% (46–49.5 min), 90% isocratic (49.5–52 min), 90–5% (52–52.7 min), and 5% isocratic (52.7–59 min).

The determination was conducted at a flow rate of 0.85 ml min^−1^ and at wavelengths of 280, 320, 370, and 520 nm. The hydroxycinnamic acid derivatives and glycosides of flavonoids were tentatively identified as deprotonated molecular ions and characteristic mass fragment ions according to Schmidt et al. (2010) and Neugart et al. (2015) by HPLC-DAD-ESI-MS^n^ using a Bruker amazon SL ion trap mass spectrometer were acquired in negative ionization mode. For the identification of the peaks, data were compared to the literature of the investigated species and their relatives. In the mass spectrometer, nitrogen was used as the dry gas (10 L min^−1^, 325°C) and the nebulizer gas (40 psi) with a capillary voltage of −3,500 V. Helium was used as the collision gas in the ion trap. The mass optimization for the ion optics of the mass spectrometer for quercetin was performed at m/z 301. The MS^n^ experiments were performed in auto up to MS^3^ in a scan from m/z 200 to 2,000. Standards (catechin, chlorogenic acid, quercetin 3-glucoside, kaempferol 3-glucoside, isorhamnetin-3-glucoside and cyanidin-3-glucoside, Roth, Karlsruhe, Germany) were used for external calibration curves in a semi-quantitative approach. Results are presented as mg kg^−1^ dry weight (DW). Accuracy and precision of the method were evalutated by calculating the limit of detection (LOD) and limit of quantification (LOQ) for the standards of quercetin 3-glucoside (LOD = 0.36 μg g^−1^ DW; LOQ = 1.07 μg g^−1^ DW), kaempferol 3-glucoside (LOD = 0.29 μg g^−1^ DW; LOQ = 0.87 μg g^−1^ DW), and isorhamnetin-3-glucoside (LOD = 0.40 μg g^−1^ DW; LOQ = 1.20 μg g^−1^). Since catechin, chlorogenic acid and cyanidin-3-glucoside are highly concentrated within peach skin, LODs were not necessary. Reproducibility of the method was assessed by setting the relative standard deviation (RSD) below 5 and 25% for main and minor peaks, respectively. Accuracy was below 2% for all compounds detected.

### Statistical analysis

Data were analyzed by two-way ANOVA followed by Tukey–Kramer *post-hoc* test at the significance level *P* ≤ 0.05 in order to evaluate the effect of both UV-B irradiation and fungal infection on each phenolic subclass detected.

Furthermore, data from individual phenolic compounds were subjected to canonical discriminant analysis (CDA) and Pearson correlation to check which experimental conditions were the most effective in determining variations of phenolic concentration, and phenolics were the most discriminant when UV-B was given alone and/or in combination with infection with *M. fructicola*.

All the statistical elaborations were performed using JMP software (SAS Institute, Inc., Cary, NC).

## Results

Since *M. fructicola* normally infects peach fruit by penetrating the skin through mechanical damages, both infected and uninfected (control) fruit were wounded and inoculated with either conidia suspension or sterile water, respectively. For this reason, possible effects of wounding, in addition to UV-B effects and/or fungal effects, were examined separately in the regions near and far from the wound. To isolate the effect of UV-B from the wounding-induced response, the region far from the wound was collected also in uninfected fruit and considered as “only UV-B treated” samples.

### Phenolic compounds

**Figure 1:**
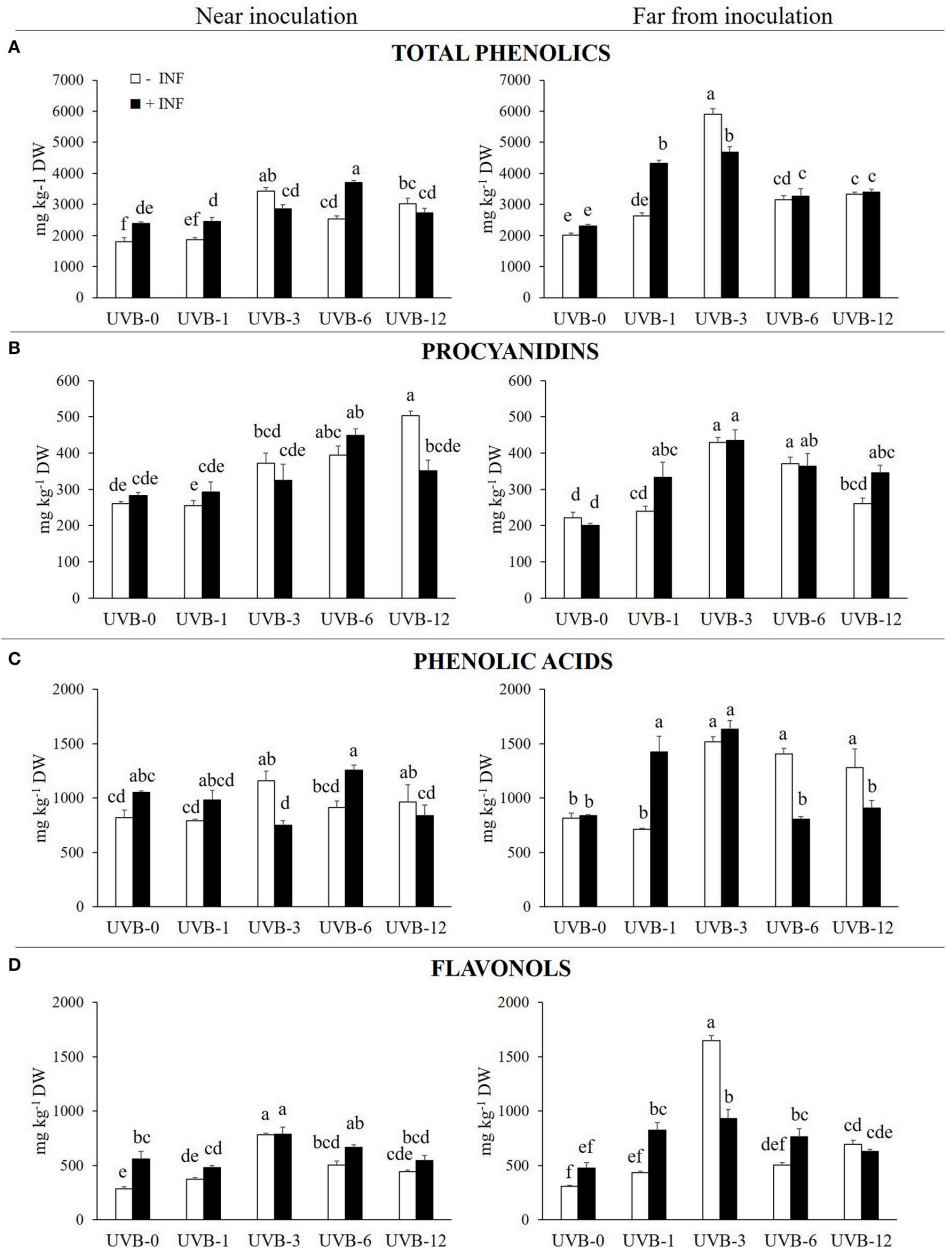
**(A)** Total phenolics, **(B)** procyanidins, **(C)** phenolic acids and **(D)** flavonols (mg kg^−1^ DW) in the skin of “Fairtime” peaches, near and far from the *M. fructicola* inoculation site, exposed to 0 h (UVB-0), 1 h (UVB-1), 3 h (UVB-3), 6 h (UVB-6), and 12 h (UVB-12) of UV-B radiation. Different letters indicate significantly different values according to one-way ANOVA (*P* ≤ 05) followed by Tukey's test.

In peach fruit a diversity of phenolic compounds has been identified and quantified including procyanidins, phenolic acids, flavonols, and anthocyanins (Table 1). Results about the phenolic subclasses are presented in Figure 1 and the concentration of each individual phenolic compound is reported in Table 2. Phenolic compounds, which represent the sum of all the phenolics detected in this work, resulted to be significantly affected by infection, UV-B and their combination both near and far from the infection site (Table 2 and Figure 1A).

**Table 1:** Phenolic compounds identified through HPLC-DAD-ESI-MS^n^ method.

**Phenolic class**	**Phenolic Compound**
**PROCYANIDINS**	
	Procyanidin dimer
	Procyanidin trimer
**PHENOLIC ACIDS**	
	Chlorogenic acid
	Neochlorogenic acid
**FLAVONOLS**	
	Quercetin-3-rutinoside
	Quercetin-3-galactoside
	Quercetin-3-glucoside
	Kaempferol-3-rutinoside
	Kaempferol-3-galactoside
	Isorhamnetin-3-rutinoside
	Isorhamnetin-3-galactoside
	Isorhamnetin-3-glucoside
**ANTHOCYANINS**	
	Cyanidin-3-glucoside

**Table 2:** Individual phenolics belonging to procyanidins, phenolic acids, flavonols and anthocyanins subclasses (mg kg^−1^ DW) in the skin of “Fairtime” peaches, near and far from the M. fructicola inoculation site, exposed to 0 h (UVB-0), 1 h (UVB-1), 3 h (UVB-3), 6 h (UVB-6) and 12 h (UVB-12) of UV-B radiation.

				**UVB-0**	**UVB-1**	**UVB-3**	**UVB-6**	**UVB-12**
**Near**								
**Procyanidins^a^**								
		Procyanidin dimer	–inf	114 ± 1 e	102 ± 3 e	178 ± 30 cde	293 ± 25 ab	346 ± 5 a
			+inf	109 ± 7 e	153 ± 24 cde	139 ± 30 de	244 ± 9 bc	213 ± 29 bcd
		Procyanidin trimer	–inf	146 ± 6 bcd	153 ± 14 bcd	195 ± 30 ab	102 ± 3 d	157 ± 15 abc
			+inf	174 ± 2 abc	140 ± 14 cd	186 ± 17 abc	205 ± 13 a	138 ± 8 cd
**Phenolic Acids^b^**								
		Chlorogenic acid	–inf	731 ± 64 cd	681 ± 12 cd	1040 ± 90 ab	822 ± 58 bcd	1033 ± 43 ab
			+inf	969 ± 22 abc	875 ± 92 abcd	638 ± 38 d	1134 ± 42 a	735 ± 96 cd
		Neochlorogenic acid	–inf	88 ± 8 cd	111 ± 5 abc	118 ± 1 ab	92 ± 11 bcd	101 ± 9 abcd
			+inf	81 ± 5 d	105 ± 5 abcd	114 ± 1 abc	124 ± 3 a	102 ± 2 abcd
**Flavonols^c,d,e^**								
		quercetin-3-rutinoside ^c^	–inf	47 ± 4 e	58 ± 7 de	120 ± 4 ab	51 ± 5 de	107 ± 2 abc
			+inf	71 ± 3 cde	67 ± 9 cde	146 ± 15 a	120 ± 9 ab	88 ± 15 bcd
		quercetin-3-galactoside ^c^	–inf	54 ± 8 c	78 ± 4 bc	212 ± 11 a	143 ± 21 b	89 ± 5 bc
			+inf	144 ± 28 b	110 ± 9 bc	229 ± 20 a	130 ± 10 b	101 ± 3 bc
		quercetin-3-glucoside ^c^	–inf	54 ± 7 e	93 ± 8 de	246 ± 16 a	127 ± 11 cde	79 ± 3 e
			+inf	128 ± 24 cde	96 ± 7 de	209 ± 21 ab	194 ± 6 abc	164 ± 32 bcd
		kaempferol-3-rutinoside ^d^	–inf	22 ± 2 c	25 ± 1 bc	31 ± 1 abc	24 ± 1 bc	28 ± 1 abc
			+inf	23 ± 3 c	36 ± 1 a	38 ± 5 a	34 ± 1 ab	32 ± 1 abc
		kaempferol-3-galactoside ^d^	–inf	3 ± 1 d	5 ± 1 cd	14 ± 1 a	6 ± 1 cd	8 ± 1 bc
			+inf	10 ± 1 b	5 ± 1 cd	8 ± 1 bc	7 ± 1 bcd	7 ± 1 bcd
		isorhamnetin-3-rutinoside ^e^	–inf	68 ± 6 c	80 ± 4 bc	80 ± 4 bc	95 ± 2 abc	76 ± 9 c
			+inf	98 ± 16 abc	125 ± 9 a	99 ± 8 abc	120 ± 15 ab	103 ± 3 abc
		isorhamnetin-3-galactoside ^e^	–inf	24 ± 1 abc	22 ± 1 c	37 ± 4 ab	27 ± 5 abc	27 ± 2 abc
			+inf	23 ± 3 bc	26 ± 2 abc	30 ± 4 abc	37 ± 1 a	33 ± 3 abc
		isorhamnetin-3-glucoside ^e^	–inf	12 ± 2 c	13 ± 1 c	43 ± 9 ab	29 ± 2 bc	27 ± 3 bc
			+inf	06 ± 7 a	16 ± 2 c	29 ± 2 bc	25 ± 1 c	19 ± 2 c
**Anthocyanins^f^**								
		cyanidin-3-glucoside	–inf	432 ± 49 d	439 ± 64 d	1108 ± 46 ab	717 ± 16 cd	950 ± 173 bc
			+inf	498 ± 4 d	695 ± 15 cd	996 ± 41 abc	1.331 ± 100 a	992 ± 58 abc
**Far**								
**Procyanidins^a^**
		Procyanidin dimer	–inf	78 ± 2 de	88 ± 5 cde	170 ± 20 abc	144 ± 1 abcd	51 ± 1 e
			+inf	69 ± 6 de	105 ± 41 bcde	211 ± 23 a	182 ± 23 ab	158 ± 15 abcd
		Procyanidin trimer	– inf	144 ± 14 cd	151 ± 13 cd	259 ± 20 a	227 ± 7ab	209 ± 16 ab
			+inf	132 ± 6 cd	229 ± 2 ab	224 ± 8 ab	181 ± 10 bcd	187 ± 8 bc
**Phenolic Acids^b^**								
		Chlorogenic acid	–inf	721 ± 45 c	597 ± 4 c	1320 ± 42 a	1304 ± 49 a	1154 ± 168 ab
			+inf	734 ± 17 c	1269 ± 150 a	1464 ± 85 a	673 ± 21 c	793 ± 71 bc
		Neochlorogenic acid	–inf	93 ± 4 e	114 ± 12 de	197 ± 12 a	103 ± 3 de	125 ± 4 cd
			+inf	104 ± 3 de	156 ± 4 bc	171 ± 10 ab	132 ± 4 cd	114 ± 1 de
**Flavonols^c,d,e^**								
		quercetin-3-rutinoside ^c^	–inf	59 ± 10 d	95 ± 3 bcd	192 ± 18 a	124 ± 22 bc	130 ± 9 bc
			+inf	86 ± 13 d	103 ± 12 bcd	119 ± 10 bc	138 ± 1 b	86 ± 1 cd
		quercetin-3-galactoside ^c^	–inf	57 ± 7 e	79 ± 5 de	496 ± 13 a	102 ± 3 de	179 ± 15 cd
			+inf	101 ± 11 de	250 ± 27 bc	304 ± 50 b	174 ± 27 cd	156 ± 16 cde
		quercetin-3-glucoside ^c^	–inf	64 ± 7 d	94 ± 3 d	600 ± 65 a	78 ± 8 d	170 ± 17 bcd
			+inf	128 ± 13 cd	242 ± 24 bc	274 ± 32 b	170 ± 27 bcd	173 ± 2 bcd
		kaempferol-3-rutinoside ^d^	–inf	18 ± 1 d	25 ± 1 cd	55 ± 2 a	34 ± 3 bc	32 ± 1 bc
			+inf	19 ± 3 d	33 ± 2 bc	31 ± 1 bc	31 ± 2 bc	37 ± 2 b
		kaempferol-3-galactoside ^d^	–inf	5 ± 1 b	4 ± 1 b	36 ± 4 a	4 ± 1 b	6 ± 1 b
			+inf	7 ± 1 b	7 ± 1 b	9 ± 1 b	08 ± 2 b	8 ± 1 b
		isorhamnetin-3-rutinoside ^e^	–inf	67 ± 6 c	92 ± 7 bc	128 ± 7 ab	111 ± 8 bc	107± 5 bc
			+inf	87 ± 18 bc	108 ± 7 bc	108 ± 15 bc	165 ± 21 a	103 ± 2 bc
		isorhamnetin-3-galactoside ^e^	–inf	17 ± 1 de	27 ± 2 cd	46 ± 7 a	29 ± 1 cd	32 ± 2 bc
			+inf	13 ± 3 e	36 ± 2 abc	44 ± 2 ab	38 ± 2 abc	33 ± 3 bc
		isorhamnetin-3-glucoside ^e^	–inf	18 ± 3 b	16 ± 2 b	92 ± 15 a	18 ± 1 b	36 ± 5 b
			+inf	35 ± 2 b	44 ± 6 b	44 ± 4 b	39 ± 9 b	35 ± 4 b
**Anthocyanins^f^**								
		cyanidin-3-glucoside	–inf	672 ± 62 e	1,242 ± 86 bcde	2,302 ± 93 a	871 ± 63 de	1,104 ± 100 cde
			+inf	796 ± 22 de	1,748 ± 178 ab	1,679 ± 263 abc	1,333 ± 168 bcd	1,507 ± 138 bc

*Accuracy and precision of the method were evalutated by calculating the limit of detection (LOD) and limit of quantification (LOQ) for the standards of quercetin 3-glucoside (LOD = 0.36 μg g^−1^ DW; LOQ = 1.07 μg g^−1^ DW), kaempferol 3-glucoside (LOD = 0.29 μg g^−1^ DW; LOQ = 0.87 μg g^−1^ DW) and isorhamnetin-3-glucoside (LOD = 0.40 μg g^−1^1 DW; LOQ = 1.20 μg g^−1^1). LODs were not necessary for catechin, chlorogenic acid and cyanidin-3-glucoside, being highly concentrated within peach skin. Reproducibility of the method was assessed by setting the relative standard deviation (RSD) below 5% and 25% for main and minor peaks, respectively. Accuracy was below 2% for all compounds detected*.

*Mean value (n = 3) ± standard error. For each metabolite values followed by different letters are significantly different according to one-way ANOVA (P ≤ 05) followed by Tukey's test*.

a *Procyanidins quantified as catechin*.

b *Hydroxycinnamic acids quantified as chlorogenic acid*.

c *Flavonols quantified as quercetin-3-glucoside*.

d *Kaempferol-3-glucoside*.

e *Isorhamnetin-3-glucoside*.

f *Anthocyanins quantified as cyanidin-3-glucoside*.

When the region close to the wound was considered, the samples treated solely with UV-B radiation underwent an increase in phenolics concentration with exposure times of 3 h or more. Particularly, the maximum of phenolics detected (3,421 mg kg^−1^ DW) was reached with UVB-3 treatment. In the region far from the wound, the trend of phenolic accumulation was similar to the region near the wounds, if we consider the uninfected samples. In fact, the UV-B radiation positively affected the phenolic concentration in UVB-3-, UVB-6-, and UVB-12-treated samples, with its maximum in UVB-3 exposed fruit (5,895 mg kg^−1^ DW).

The fungus itself did not affect the phenolic concentration far from the necrotic area at UVB-0. However, it induced a significant increase of phenolics (33%) near the infection.

When UV-B was given prior the fungus, the phenolic concentration near the infection was not affected by any of the UV-B pre-treatment except for UVB-6, where the exposure increased phenolics by 55% compared to infected UVB-0. However, when the UV-B-pre-treated fruit are compared with the uninfected ones, UVB-1 and UVB-6 had an additive effect on phenolics concentration (by 21 and 47%, respectively) while UVB-3 had a subtractive effect (−16%). No differences were detected between infected and uninfected UVB-12 samples. The most stimulating treatments for phenolic accumulation far from the necrotic area were UVB-1 (87%) and UVB-3 (102%), although a significant increase was detected also with UVB-6 (41%) and UVB-12 (47%) when compared to infected and UVB-0-treated samples. When results of the combined factors are compared with the correspondent uninfected ones, UVB-1 treatment had an additive effect (+65%) on the phenolic accumulation observed without the fungus, while UVB-3 had a subtractive effect (−21%).

These results suggest a time-dependendent priming effect of UV-B exposure pre-treatment for *M. fructicola* infection.

### Procyanidins

The procyanidins concentration was calculated as the sum of procyanidin dimer and trimer, quantified individually. Effect on procyanidins accumulation was different according to the sampled peach area, near or far from the wound/necrotic area (Figure 1B).

Near to the not-inoculated wounds, procyanidins concentration increased proportionally with the UV-B dose given, reaching a concentration of 503 mg kg^−1^ DW in the UVB-12 samples. If the region far from the inoculation point is taken into account, the UV-B radiation determined an increase of the procyanidins with UVB-3 (93%) and UVB-6 (67%) doses in uninoculated samples (Figure 1B).

The procyanidins were affected by the presence of *M. fructicola* only far from the necrotic area, and both the sampled region resulted to be influenced by either the UV-B treatment or the combined pre-UV-B/inoculation treatment (Table 3).

**Table 3:** The two-way ANOVA *P*-values for the effect of infection, UV-B treatment, and of their interactions.

		**Two-way ANOVA (*****P*****)**		
		**Infection**	**UV-B**	**Infection x UV-B**
Total phenolics	Near	0.0002^***^	<0.0001^***^	<0.0001^***^
	Far	0.0320^*^	<0.0001^***^	<0.0001^***^
Procyanidins	Near	0.2707	<0.0001^***^	<0.0004^***^
	Far	0.0408^*^	<0.0001^***^	0.0437^*^
Phenolic acids	Near	0.7581	0.0332^*^	<0.0001^***^
	Far	0.6494	<0.0001^***^	<0.0001^***^
Flavonols	Near	<0.0001^***^	<0.0001^***^	0.0179^*^
	Far	0.8886	<0.0001^***^	<0.0001^***^
Cyanidin-3-glucoside	Near	0.0005^***^	<0.0001^***^	<0.0001^***^
	Far	0.0464^*^	<0.0001^***^	0.0005^***^

*A single asterisk indicates significance at P ≤ 0.05, two asterisks at P ≤ 0.01, and three asterisks at P ≤ 0.001*.

Due to the inoculation with fungus after the UV-B treatment, in the region close to the infection the procyanidins concentration increased significantly only with UVB-6, in which the inoculated fruits showed the highest procyanidin concentration (448.8 mg kg^−1^ DW) among all the inoculated ones. If the uninfected and the infected peaches were compared, they did not differ in terms of procyanidin concentration except for the UVB-12 treatment, where the uninfected ones displayed a significantly higher concentration compared to the corresponding infected fruits (43%).When the individual procyanidins are considered, however, the behavior is different between the procyanidin dimer and the trimer (Table 2). The procyanidin dimer in infected samples resulted to be enhanced only for the highest doses tested, UVB-6 and UVB-12, when compared to the infected UVB-0 samples. On the contrary, when the infected and uninfected fruit are compared considering each UV-B pre-treatment, no differences were detected for any UV-B dose except for UVB-12, where the combination of the two factors resulted in a significant decrease of procyanidin dimer. Regarding procyanidin trimer, the UV-B pre-exposure did not alter the fungus-induced response for any of the UV-B pre-treatment. Far from the necrotic area, the infected samples displayed a significant accumulation of procyanidins for all the UV-B-dose given, with the maximum with UVB-3 treatment (434.8 mg kg^−1^ DW). Regarding the individual procyanidinds, the procyanidin dimer exhibited the highest concentration in UVB-3, UVB-6, and UVB-12 compared to UVB-0 infected ones. Generally, the combination of UV-B and fungus resulted in an additive effect for all the UV-B pre-exposures, although such increase was significant only for UVB-12. Contrarily, procyanidin trimer concentration was enhanced in UVB-1, UVB-3, and UVB-12 in comparison to UVB-0 infected fruit. The two factors did not result in altering procyanidin trimer content compared to the correspondent uninfected peaches except for UVB-1, where the combination of the factors had an additive effect on the concentration of such procyanidin.

### Phenolic acids

Regarding the phenolic acids, the values represent the sum of chlorogenic and neochlorogenic acids concentration for each replicate. Their concentration was affected only by UV-B radiation and interaction between infection and UV-B (Table 3).

Near the wound, the UV-B treatment itself induced a significant accumulation in phenolic acids concentration only considering the UVB-3 (41%) and UVB-12 (17%) doses (Figure 1C). Far from the wound, the UV-B radiation significantly induced an accumulation of phenolic acids in all the UV-B treatments longer than 1 h.

The infection itself did not display any significant modification in phenolic acids concentration, in both near and far regions from the necrotic area.

However, when UV-B was given prior fungal inoculation, 3 and 12 h UV-B doses showed a significant decrease by 35 and 13%, respectively, compared to the corresponding uninoculated samples near the necrotic area. UVB-3 samples also showed a decrease (by 28%) when compared to the inoculated but unirradiated sample. The remaining UV-B treatments, on the contrary, did not show any variation compared to the infected-UVB-0 control. However, an increase in phenolic acids concentration far from the necrosis was detected only with 1 and 3 h of UV-B pre-treatments, by 70 and 95%, respectively, compared to the infected UVB-0 samples. Considering the individual phenolic acids detected, the behaviors described above for both the far and near regions can be particularly valid for chlorogenic acid, mainly because it is 10-times more concentrated than neochlorogenic acid. The neochlorogenic acid concentration near the infection resulted to be increased by UVB-3 and UVB-6 pre-treatments, compared with UVB-0 infected samples. No differences were detected between infected and uninfected samples considering each UV-B pre-exposure, except for UVB-6, where the infection had a significant additive effect to the UVB-6 treatment. Far from the infection, as observed for the total phenolic acids, the neochlorogenic acid was enhanced by UVB-1 and UVB-3 pre-treatments.

### Flavonols and cyanidin-3-glucoside

Flavonols are considered as the sum of all the individual flavonols detected and listed in Tables 1, 2. Flavonols displayed a similar behavior near and far from the infection, although the entity was different (Figure 1D).

In fact, the UV-B radiation determined a significant increase in flavonols concentration in the UV-B-irradiated skin for UVB-3 and UVB-6 by 174 and 76%, respectively, in comparison with uninoculated UVB-0-treated samples. Moreover, in the uninfected peaches, the maximum in flavonols concentration was observed with 3 h of UV-B radiation in both near and far from the wound, reaching values of 782 and 1.646 mg kg^−1^ DW, respectively.

When the fungus was inoculated in the fruit, without UV-B irradiation, the infection determined a significant accumulation (95%) of flavonols close to the necrotic area. However, far from the infection, no significant difference were detected between the inoculated and uninoculated samples (Table 3).

When peaches were pre-treated with UV-B and then inoculated with the fungus, for all the UV-B exposures tested the flavonols concentration was similar to the corresponding uninfected samples near the necrotic area. However, when compared with the un-irradiated samples, UVB-3 was the only pre-treatment affecting positively (+41%) the flavonols level. Among the detected flavonols, the effectiveness of the UVB-3 pre-treatment was observed especially for quercetins, which all displayed a significant peak in correspondence to UVB-3 exposure. However, for all of them, such increase was observed already in the UVB-3 without infection, underlying the overwhelming effect of UV-B-induced over the infection-induced effects. Kaempferols, which are the less concentrated flavonols among the ones found, behaved differently between each other. While kaempferol-3-rutinoside exhibited a significant enhancement with UVB-1 and UVB-3 pre-treatments, both compared to the correspondent uninfected and the UVB-0 infected groups, the kaempferol-3-glucoside showed no effect of the UV-B pre-exposure for all the UV-B doses except for UVB-3, where the combination of UV-B and infection negatively affected its concentration compared to the only UV-B-treated one. Isorhamnetin-3-rutinoside concentration, for any pre-treatment, did not change compared to the un-exposed samples. However, the infection following UV-B exposure showed a tendency to increase its concentration for any UV-B pre-exposure compared to the uninfected ones, although it was significant only for UVB-1.

Far from the necrosis, however, the flavonols concentration was significantly higher compared to the UVB-0 infected group for all the UV-B treatments except for UVB-12 (72% for UVB-1; 96% for UVB-3; 60% for UVB-6). However, when compared to the correspondent uninfected samples for each UV-B dose, the infection resulted to positively enhance flavonols concentration only in UVB-1 and UVB-6 (+90 and +52%, respectively), while in UVB-3 the combination of the two factors drastically decreased its concentration (-43%). In UVB-12 pre-treated group, no changes were detected compared to UVB-12 uninfected samples. Since quercetins were the most abundant flavonols detected, the trend observed for the total flavonols is mainly due to the quercetins behavior. They all exhibited a significant peak at UVB-3, compared to UVB-0 infected group, although the presence of the fungus significantly decrease their concentration in comparison to the UVB-3 uninfected ones. While UVB-12 was not effective for all the quercetins, UVB-1 determined an increase only in the quercetin-3-galactoside, while UVB-6 only in the quercetin-3-rutinoside, compared to the UVB-0 infected group. Regarding kaempferols, only kaempferol-3-rutinoside was positively affected by the UV-B pre-treatment, regardless the UV-B dose, while kaempferol-3-galactoside did not change at all when compared to the UVB-0 infected samples. As observed for quercetins, the combination of UV-B and infection impacted negatively kaempferols concentration especially in UVB-3, which was the most effective UV-B dose to stimulate flavonols content without infection. Concerning isorhamnetins, the isorhamnetin-3-galactoside was the most responsive one toward the pre-UV-B exposure and the inoculation, since it increased for any UV-B dose tested. However, since no significant differences were detected between infected and uninfected samples for any UV-B duration, the increase observed was likely due to the UV-B treatment over the combination of pre-UV-B and infection. The isorhamnetin-3-glucoside showed no changes between the UVB-0 and any of the UV-B pre-treatment, although the presence of the fungus in UVB-3 group drastically decreased isorhamnetin-3-glucoside concentration in comparison to the UVB-3 uninfected group. Similarly, isorhamnetin-3-rutinoside was not responsive to the combination of UV-B pre-exposure and infection except for UVB-6 dose, where the concentration was significantly higher compared to both the UVB-0 infected and the UVB-6 uninfected groups.

Cyanidin-3-glucoside was the only anthocyanin detected in peach skin (Tables 1, **2**). Its concentration was significantly affected by both factors (infection and UV-B) and their interaction (Table 3).

The UVB-3 and UVB-12 were the only effective treatments in stimulating its accumulation near the wound in the uninfected samples, with an increase of 156 and 120%, respectively. Far from the inoculation site, similar to what observed near the wound, the cyanidin-3-glucoside concentration reached the maximum in the UVB-3 treatment (2,302 mg kg^−1^ DW). However, all the remaining UV-B treatments resulted to be not significant compared to UVB-0.

The presence of the fungus in UVB-0 samples did not induce any significant change in terms of cyanidin-3-glucoside concentration either near or far from the necrotic area.

In the presence of *M. fructicola*, the significantly effective UV-B exposure were UVB-3, -6, and -12, increasing the cyanidin concentration by 100, 167, and 99%, respectively, compared to the infected UVB-0 samples near the necrosis. However, far from the infection, all the UV-B pre-treatments were found to be effective in increasing the concentration of such anthocyanin.

### Canonical discriminant analysis (CDA) and pearson correlation

**Figure 2:**
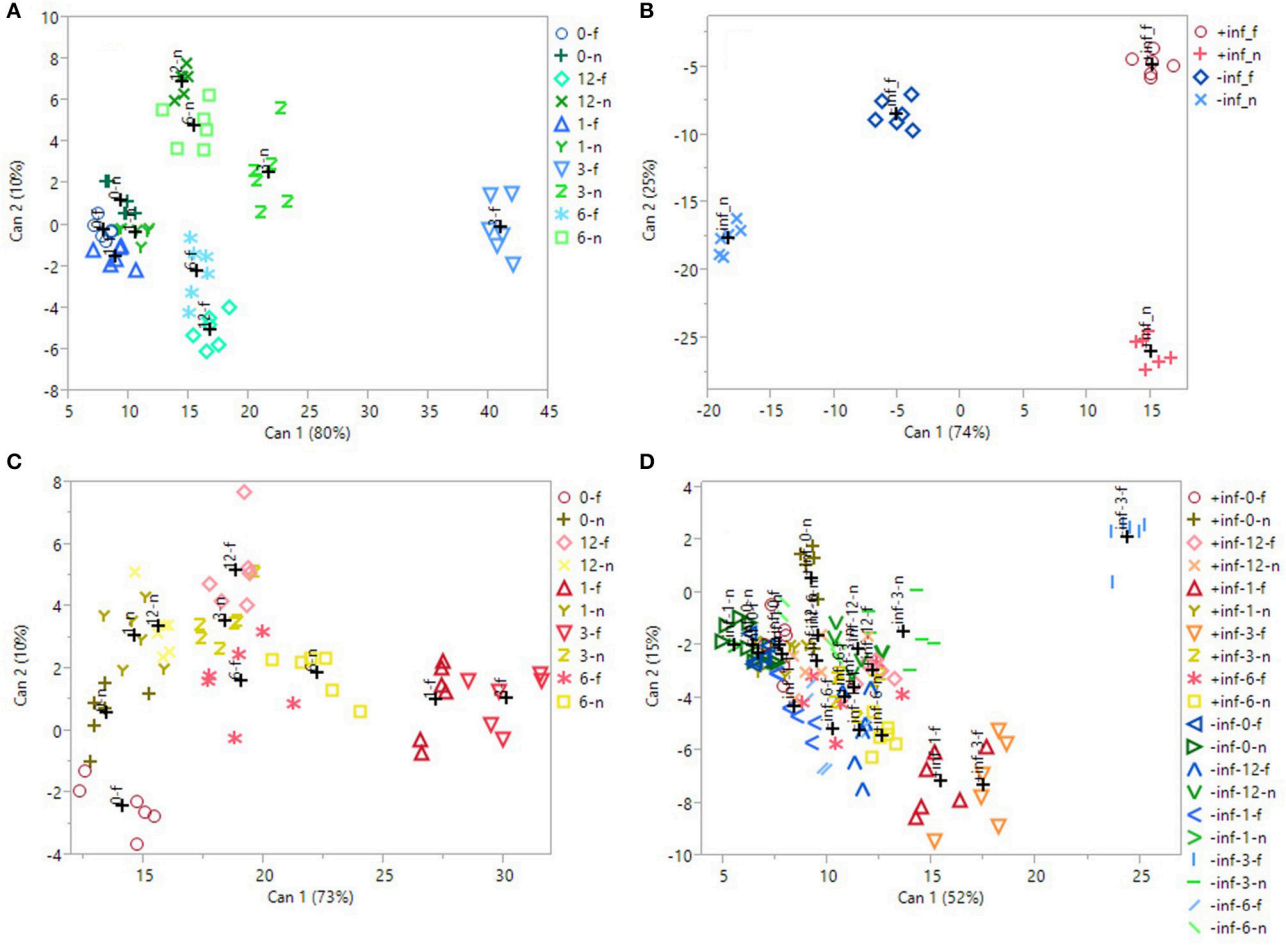
2D scatterplot of canonical discriminant analysis considering **(A)** the uninfected, UV-B-treated samples; **(B)** the UV-B-untreated samples; **(C)** the infected, UV-B-treated samples; **(D)** all the samples. In **(A,C)**, 0/1/3/6/12_f/n refers to the samples irradiated with UV-B for 0/1/3/6/12 h, considering the region far/near the inoculation point. In **(B)**, ±inf_f/n refers to the infected/uninfected samples, considering the region far/near the inoculation point. In **(D)**, ±inf_0/1/3/6/12_f/n refers to the infected/uninfected samples, irradiated with UV-B for 0/1/3/6/12 h, considering the region far/near the inoculation point. Can 1 and 2 refers to the canonical function 1 and 2, which considers all the variables in order to maximize the separation among the groups.

Through canonical discriminant analysis (CDA), it is possible to determine whether the biological replicates fit preassigned groups according to all the variables measured. Four different CDAs were performed (Figure 2) to check the effectiveness of UV-B and/or fungal infection in separating the groups. Four MANOVA tests (Wilk's Lambda, Roy's Largest Root Test, the Hotelling-Lawley Trace, and the Pillai-Bartlett Trace) were also implemented for each CDA, to check whether the groups were different according to the variables given. For each CDA, all the MANOVA test gave very significant results (*P* < 0.0001), indicating the robustness of the groups separation for each CDA. Furthermore, the Pearson coefficients were determined for each CDA between the concentrations of each phenolic compound and the respective canonical score, to find out which individual phenolics were the main responsible for the groups separation.

The first CDA was performed on just the uninfected samples (Figure 2A), to test the effectiveness of the UV-B exposure itself. Canonical function 1 explained the majority of the separation (80%), and the best segregation was visible for the UVB-3 group, both near and far from the wound, especially for the last one. All the other groups overlapped with each other on the left portion of the plot. The Pearson correlation (Table 4) revealed that all the phenolics identified in this study were discriminant in this CDA, except of procyanidin dimer.

**Table 4:** Pearson's correlation coefficients (r) between each phenolic compound detected and the canonical scores for each canonical discriminant analysis (CDA) reported in Figure 2.

	**Pearson coefficient**			
	**CDA A**	**CDA B**	**CDA C**	**CDA D**
**PROCYANIDINS**				
Procyanidin dimer	0.19	−0.35	0.25	0.23
Procyanidin trimer	0.63^*^	0.15	0.74^*^	0.69^*^
**PHENOLIC ACIDS**				
Chlorogenic acid	0.62^*^	0.42	0.68^*^	0.62^*^
Neochlorogenic acid	0.79^*^	0.12	0.90^**^	0.82^**^
**FLAVONOLS**				
quercetin-3-rutinoside	0.75^*^	0.55	0.37	0.67^*^
quercetin-3-galactoside	0.97^**^	0.62^*^	0.66^*^	0.89^**^
quercetin-3-glucoside	0.93^**^	0.71^*^	0.69^*^	0.88^**^
kaempferol-3-rutinoside	0.90^**^	0.02	0.23	0.71^*^
kaempferol-3-galactoside	0.92^**^	0.87^**^	0.09	0.78^*^
isorhamnetin-3-rutinoside	0.60^*^	0.37	0.07	0.30
isorhamnetin-3-galactoside	0.70^*^	−0.30	0.67^*^	0.69^*^
isorhamnetin-3-glucoside	0.85^**^	0.78^*^	0.17	0.70^*^
**ANTHOCYANINS**				
cyanidin-3-glucoside	0.83^**^	0.43	0.74^*^	0.82^**^

*CDA A refers to the uninfected and UV-B-treated samples; CDA B refers to just the UV-B-untreated samples (UVB-0); CDA C refers just to the infected and UV-B-treated samples; CDA D refers to all the samples. For each CDA, only canonical scores from canonical function 1, which explains 80% (CDA A), 74% (CDA B), 73% (CDA C) and 52% (CDA D) of the respective separation*.

* *0.6 > |r| > 0.8: strong correlation*.

** *0.8 > |r| > 1: very strong correlation*.

In Figure 2B, only the samples that did not receive any UV-B treatment were considered (UVB-0), to test whether the presence of the fungus, as well as the wounding in uninfected samples, induced an effect in altering phenolic concentration. The CDA were found to be very successful in discriminating the groups, with 74% of segregation explained by canonical function 1, and 25% by canonical function 2. Particularly, the major classification on canonical function 1 is between the infected samples, regardless the sampling region, and the uninfected ones. Furthermore, a clear separation is visible also between the near and far samples of the uninfected ones. According to Pearson correlation (Table 4), the most discriminant compounds were all flavonols. Particularly, the strongest correlation was found for kaempferol-3-galactoside, followed by the quercetin-3-galactoside, the quercetin-3-glucoside and the isorhamnetin-3-glucoside.

Later, the real effect of the pre-UV-B-irradiation in combination with following *M. fructicola* infection was investigated (Figure 2C). The 3 h-UV-B exposure resulted to be well-separated also among the samples UV-B-treated and inoculated with *M. fructicola*. In fact, in the CDA including only the fruit that have received both the stressors combined (Figure 2C), the UVB-3 treatment, far from the infection, resulted to induce the most remarkable effect in altering phenolics concentration according to canonical function 1 (73% of separation). Immediately after UVB-3 group, UVB-1 one was also well-clustered on the right portion of the plot, supporting the idea of a positive effect of both UVB-3 and UVB-1 exposures on phenolic accumulation. As observed for uninfected samples (Figure 2A), also for infected samples (Figure 2C) all the other groups are not well-separated and are located almost indistinguishably in the left half of the scatterplot. Interestingly, for both the last CDAs, the UVB—treated groups are the leftmost groups, suggesting that the UV-B treatment, regardless the duration, had a positive effect in increasing phenolics concentration. The Pearson correlation (Table 4) indicates as strongly correlated compounds the cyanidin-3-glucoside, the isorhamnetin-3-galactoside, the quercetin-3-glucoside, the quercetin-3-galactoside, the procyanidin trimer, the chlorogenic acid and the neochlorogenic acid.

Finally, in Figure 2D, all the samples, regardless the UV-B exposure time, the sampling region or the infection, have been considered for the CDA. Although canonical function 1 explains 52% of the separation, the CDA showed only a partial separation among the groups. In fact, most of the groups are overlapped and located in the left half of the scatterplot. This could be due to overlapping effects of both UV-B treatments, that might be too strong or too weak, and the infection process. However, it is possible to see a good segregation of the uninfected UVB-3 group far from the wound, on the top-right edge of the plot. Other well-separated groups, still on the most positive half of the hyperspace, are the infected UVB-1 and UVB-3 far from the infection, and the uninfected UVB-3 near the wound. The separation of these groups from the others were due to most of the phenolics identified in this study except for the procyanidin dimer and the isorhamnetin-3-rutinoside (Table 4).

## Discussion

Using an HPLC-DAD-MS^n^ system it was possible to detect many phenolic compounds that belong to several phenolic subclasses, such as procyanidins (procyanidin dimer and trimer), phenolic acids (chlorogenic and neochlorogenic acid), flavonols (quercetin-3-rutinoside. quercetin-3-galactoside, quercetin-3-glucoside, kaempferol-3-rutinoside, kaempferol-3-galactoside, isorhamnetin-3-rutinoside, isorhamnetin-3-galactoside, isorhamnetin-3-glucoside, cyanidin-3-glucoside), and anthocyanins (cyanidin-3-glucoside). All of these compounds have previously been identified in peach fruit (Scattino et al., 2014).

Our aim was to investigate whether the phenolic response to *M. fructicola* infection might be enhanced by a UV-B pre-treatment. For this reason, since this pathogen normally infects peach fruit by penetrating the skin through mechanical damages, both infected and uninfected (control) fruit were wounded and inoculated with either conidia suspension or sterile water, respectively.

Both single UV-B treatment and in combination with a following infection with *M. fructicola*, resulted in significant modulation of phenolic concentration in peach skin, with a differential behavior according to each phenolic compound considered.

When UV-B radiation was given without the infection, 3 h of UV-B exposure seemed to be the best UV-B dose to obtain the maximum phenolic accumulation. This effect, particularly visible on total phenolics, is reflected mainly by flavonols, phenolic acids, cyanidin-3-glucoside and procyanidins far from the wound. UV-B-induced increase in the concentration of phenolic compounds, especially flavonoids has been previously described (Schreiner et al., 2014). Recent evidences in literature suggest that UV-B radiation influences phenolics concentration in peach skin in accordance to the phenolic compound/subclass considered (Scattino et al., 2014; Santin et al., 2018). Indeed, Santin et al. (2018) found that a 10 and 60 min UV-B treatment determines an increase of specific phenolic compounds, such as anthocyanins, flavones and dihydroflavonols, after 36 h from the treatment. In Scattino et al. (2014), similarly, a different behavior was found between hydroxycinnamic acids and flavonols in response to UV-B radiation in different peach cultivars. In the present study, a UV-B treatment longer than 3 h might cause the activation of generic stress-induced intracellular pathways which might have overlapped the UVR8-mediated signal. Indeed, it might be that a UV-B exposure extended over 3 h have led to an excessive production of reactive oxygen species (ROS), causing the degradation/consumption of the phenolics within the cell in order to avoid potential damages to macromolecules. In fact, the total flavonols decreased of about 36 and 44%, respectively, at 6 and 12 h of UV-B irradiation compared to UVB-3. The UV-B-triggered production of ROS has been observed in tobacco leaves (Czégény et al., 2014), where it was found that UV-B radiation is capable of forming hydroxyl radicals from hydrogen peroxide. Moreover, strong and prolonged UV-B radiations were found to activate generic stress pathways in *Arabidopsis* leaves, thus not inducing phenolic accumulation as specific acclimation effect which are instead favored by short (1–6 h) and mild UV-B exposures (Favory et al., 2009; Jenkins, 2017). The canonical discriminant analysis considering only the UV-B-treated but uninfected fruit (Figure 2C) also indicates UVB-3 treatment as the most effective one in stimulating phenolics accumulation, since they are the first two groups on the right side of the plot. Furthermore, regarding total phenolics, as well as for flavonols and the cyanidin-3-glucoside, the concentration detected after UVB-3 treatment was much higher far from the wound than near the wound. In fact, it might be possible that the positive effect of UV-B-radiation in stimulating phenolic accumulation is counteracted by the negative effect of the wound leading to a phenolic depression, which represented a stress for the fruit causing metabolic dysregulations in the region nearby. Another hypothesis could be that, since the region near the infection is likely already colonized by the fungus and will be quicky necrotized, the fruit triggers defense mechanisms in the health tissue (far from the fungus). The first evidence in literature that demonstrated the presence of long-distance signal molecules induced by biotic stresses, which can make plant tissues distant from the infection less susceptible to further biotic attacks, dates back in 1980 (Guedes et al., 1980). This so-called systemic acquired resistance (SAR), involves a wide set of signal molecules, such as salicylic acid, systemin, methyl jasmonate, jasmonic acid, and ethylene, which diffuse from the infection site toward undamaged plant tissues (Enyedi et al., 1992). In addition, several more hormones have been more recently found to be associated with an increased adaptability of the plants toward biotic stresses, such as abscisic acid, auxin, gibberellic acids, cytokinins, and brassinosteroids, whose signaling pathways partially overlap and stimulate the distant and healthy regions of the plant to synthesize defensive compounds to increase its survival chance from eventual pathogen spread on that tissue (Takatsuji and Jiang, 2014). However, such signaling mechanisms have been mostly investigated in plant models, thus knowledge about the pathogen-induced migration of defensive molecules on fruit is scarce. in Among such defense mechanisms, the accumulation of anti-fungal phenolics might also be crucial to limit the infection spreading. The wound effect is visible also through the canonical discriminant analysis (Figure 2A), in which the uninfected samples near and far from the wound are clearly separated in the scatterplot considering both canonical function 1 and 2. Previous works investigated the effect of mechanical wounding on fruit and vegetables. For example, it was observed that PAL activity is induced by cutting lettuce leaves in 2 × 2 cm pieces (Saltveit, 2000). However, it was found that phenolics and anthocyanins concentration, as well as the concentration of several other secondary metabolites and the antioxidant capacity, strictly depends on the plant species considered (Fernando Reyes et al., 2006). In fact, it was found that phenolic concentration decreased in zucchini, radish, potato, and red cabbage subjected to shredding process by 26, 7, 15, and 9%, respectively, while it increased in lettuce, celery, carrot, parsnips and sweet potato by 81, 30, 191, 13, and 17%, respectively (Fernando Reyes et al., 2006).

Through CDA considering only the UV-B-exposed peaches (Figure 2C), it was possible to observe that between the UVB-3-treated groups, the one sampled far from the wound resulted to be located distantly in the right region of the scatterplot compared to the corresponding group near the wound, indicating a reduction of phenolics concentration close to the wounding site. From the same CDA, it was also possible to confirm the effectiveness of the UVB-3 treatment compared with the other UV-B treatments, since the UVB-3 groups are located in the furthest right portion of the plot, considering canonical function 1.

When infection was given alone, without any UV-B exposure, it determined a modulation in phenolic profile compared to the uninfected ones. Particularly, the infection determined an increase in total phenolics and in flavonols near the infection, although slight but no significant increases were detected for several phenolic classes also far from the infection. It has been stated that phenolics, together with phytoalexins and other plant-defensive secondary metabolites, tend to accumulate in cells surrounding the infection as part of a locally induced defense response (Lattanzio et al., 2006). In this study, the fungus-induced accumulation of phenolics can be observed also in the corresponding CDA (Figure 2A), where the infected groups are highly different from the uninfected ones, positioning themselves on the right edge of the plot. Considering the far region, phenolic acids increased in UVB-3, -6, and -12, being the only phenolic subfamily analyzed that did not show a decrease for UV-B treatments longer than 3 h. Phenolic acids represent a crucial junction point in the phenylpropanoid pathway, since they are precursor of most of flavonoids, such as flavonols, anthocyanins and procyanidins. The constantly high level for such UV-B treatments might be due to either a continuous stimulation of their biosynthesis, or their reduced conversion into downstream flavonoids. Especially this last hypothesis might be supported by the fact that flavonols in UVB-6 and UVB-12 samples, as well as procyanidins in UVB-12 samples, decreased to the control level.

The individual phenolics detected that led to the segregation among the infected/uninfected samples, according to the CDA (Figure 2A), were quercetin-3-galactoside, kaempferol-3-galactoside and isorhamnetin-3-glucoside. Involvement of several phenolic subclasses in counteracting fungal infection has been observed in previous studies. Recently, a comparison between the phenolic profile of two apple cultivars, one resistant and one susceptible to blue mold caused by *Penicillium expansum*, revealed that the resistant apple cultivar had higher concentrations of procyanidins, dihydrochalocone, flavonols, and hydroxycinnamic acids (Sun et al., 2017). In bilberry (*Vaccinium myrtillus*) infected by the fungal pathogen *B. cinerea*, an accumulation of several phenolics such as quercetin-3-glucoside, quercetin-3-*O*-α-rhamnoside, quercetin-3-*O*-(4″-HMG)-R-rhamnoside, chlorogenic acid and coumaroylquinic acid was found (Koskimäki et al., 2009). The antifungal role of phenolics was observed also in nectarine and apricot fruits treated with *Sanguisorba minor* extract, where a drastic inhibition of *Monilinia laxa* brown rot was observed due to the high presence of caffeic acid derivatives and flavonoids derived from apigenin, quercetin, and kaempferol in the extract (Gatto et al., 2011).

However, under natural conditions, plants have to face different biotic and abiotic stresses simultaneously, due to their sessile lifestyle, and whose effects are not simply the sum of each individual stressor. For this reason, in this work an attempt was made to apply a combination of a pre-UV-B radiation and a fungal infection, to investigate the responsiveness of phenolic compounds.

When peaches were pre-treated with UV-B and then infected with *M. fructicola*, the scenario changed, and variations were again different according to each phenolic class and individual compound considered. In fact, the UV-B pre-exposure generally induced an accumulation of phenolics compared to the UV-B unexposed samples, especially for UVB-1 and UVB-3 treatments in the region far from the infection. This behavior is particularly visible for flavonols, phenolic acids, cyanidin-3-glucoside and, generally, total phenolics. Such phenolic increment in the far region was not visible in the UVB-1- and UVB-3-treated samples near to the infection probably because the fungus, which already spread and induced brown rot symptoms near the inoculation site, induced partial degradation/consumption of UV-B-induced peach phenolics in the area nearby. This behavior was visible also through CDA (Figure 2D), where the only groups well-separated from the others in the most positive region of the scatterplot were UVB-1- and UVB-3-treated samples far from the infection. Considering UVB-1 treatment it is noticeable that the infected samples far from the inoculation site showed a higher phenolic concentration than the corresponding UVB-1-uninfected ones. It is intriguing to note that *M. fructicola* did not induce any change in phenolic concentration unless UV-B radiation was preliminary applied. Similarly, UV-B radiation at the lowest dose (UVB-1) was ineffective in stimulating phenolic accumulation. However, when UVB-1 peaches were infected, phenolics concentration increased. This might be due to an additive effect of the systemic response toward the fungus and the UVB-1 radiation, which itself might have been too mild to induce significant phenolics accumulation. This evidence suggests that signals deriving from the individual factors, too low to induce phenolic biosynthesis, synergically interacted, thus triggering a positive response. Differently from the general trend procyanidins were unaffected by the fungus but only stimulated by UV-B treatments. Regarding UVB-3 treatment, which was the most effective in determining phenolic accumulation without infection, the inoculation of *M. fructicola* resulted in a significant decrease of flavonols and total phenolics far from the infection, while for the other phenolic subclasses no variations were detected. This suggests an impact-specific response in phenolic accumulations. Since UVB-3 was found to be the threshold dose in stimulating phenolic accumulation in absence of fungus or wounding effect, it might be that either the systemic response triggered by *M. fructicola*, or longer UV-B treatments, did not result in enhancing phenolics further. Effectiveness of UVB-3 treatment was observed especially for flavonols, which represent very strong antioxidant compounds among favonoids.

Regarding phenolic acids, it is interesting to notice that, contrarily to what observed when UV-B was given alone, UVB-6 and UVB-12 determined a significant decrease in their concentration as compared to the UVB-3 treatment in the far region. As already stated above, phenolic acids represent precursors for several phenolic subfamilies which might act as defensive compounds also against biotic stresses. Thus, it is likely that such decrease might be due to their utilization in forming antioxidant and antifungal compounds such as procyanidins or flavonols, which in fact remained similar to UVB-3 also for higher doses.

The simultaneous or subsequent presence of different stressors or changes in environmental factors sharing common responses (e.g., induction of phenolic metabolism) may result in a positive or negative effect on metabolite production, as indicated by some results present in literature. Pan et al. (2004) found that the application of UV-C radiation (1.41 kJ m^−2^) and heat (45°C, 3 h in air), resulted in decreasing phenolics content after 2-days storage at 20°C. Very few previous works investigated the phenolics response to a combined exposure to UV-B radiation and pathogen. In a work by Saijo et al. (2009), the sucrose-induced accumulation of anthocyanins was attenuated by a simultaneous application of bacterial elicitors flg22 and elf18 in Arabidopsis, suggesting a potential subtractive effect of a biotic stress on phenolics production. A previous study in literature found that genes associated with the response of *Nicotiana longisflora* plants to herbivory insect were also induced by UV-B radiation, while, in parsley, pathogen-induced defense responses could inhibit the UV-induced flavonoid biosynthesis (Logemann and Hahlbrock, 2002). Similarly, another work reports a decrease in the UV-B-induced accumulation of flavonols by concurrent application of a bacterial elicitor in *Arabidopsis* cell culture (Schenke et al., 2011). Such a suppression was accompanied by the production of defense-related compounds as phytoalexins and lignin that can limit pathogen spread acting as a structural barrier. Moreover, it is also possible to state that, in the present study, UV-B treatment was the most effective factor, over the fungal infection, in increasing phenolic concentration in peach skin. In fact, the highest phenolics concentration was reached when UV-B radiation was given without fungal infection mainly in the far region. This is particularly valid for flavonols and cyanidin-3-glucoside, which are among the most antioxidant phenolics in plant kingdom. In the CDA including all the groups considered in this study (Figure 2B), the most separated group on the right part of the plot was the uninfected and UVB-3 treated one, far from the wound. This group, among the UVB-3 groups, was the one not affected by stimuli other than UV-B radiation, given at the best dose tested. The overwhelming effect of UV-B radiation compared to the fungal infection was visible also by the Pearson coefficients (Table 4). In fact, while the presence of the fungus alone led to the increase of just four out of thirteen phenolics identified, the presence of UV-B radiation alone induced variation of 12 out of 13 phenolics, with very strong correlation values (|r| > 80) for most of flavonols and the cyanidin-3-glucoside.

No previous data are reported in literature about the effects of a pre-UV-B exposure on phenolics concentration of peach fruit inoculated with *M. fructicola*. Our study revealed that all the phenolic subclasses identified are enhanced by 1 h- and 3 h-UV-B radiations far from the inoculation point, while near the necrosis the scenario is more complex and depends on the UV-B dose applied and phenolic subclass considered, probably due to an overwhelming effect of both the fungus and the wounding. In fact, a very high conidia concentration was inoculated in this experiment to ensure the development of the infection. However, under environmental conditions, the number of conidia penetrating the fruit and giving rise to the symptoms are supposed to be much less, thus the UV-B-induced phenolic compounds might be able to counteract the fungal spreading. Specifically, in this work an accumulation of chlorogenic acid was observed both near and far from the infection following specific UV-B irradiation doses (e.g., UVB-3), both alone and in combination with the infection. Since the caffeoyl moiety of chlorogenic acid is involved in inhibiting the expression of the *Mf-cut1*, a *M. fructicola* gene encoding a cutinase enzyme, and in preventing cutinase activity as well (Bostock et al., 1999; Wang et al., 2002; Guidarelli et al., 2014), it is likely that the UV-B-induced increase in such phenolic acid might result in contrasting pathogen spreading on the fruit.

This is therefore a preliminary study, and further research is needed in order to understand whether the UV-B radiation can limit the fungal spreading under more realistic condition (inoculation with a lower conidia concentration). Furthermore, investigation on long-distance fungus-induced molecules are encouraged, to unreveal the signaling pathways involved in the phenolic response both at molecular and biochemical levels. Moreover, deeping the knowledge about the relationship between phenolic structure and their effect as protective compounds against fungal infection is strongly recommended.

## Author contributions

AR, GV, and MoS designed the research. MaS, SS, SN, AC, and MB carried out the experiments, analyzed the data, and wrote the manuscript. AR, GV, and MoS helped to draft the manuscript and revise the manuscript. All authors read and approved the final manuscript.

### Conflict of interest statement

The authors declare that the research was conducted in the absence of any commercial or financial relationships that could be construed as a potential conflict of interest.
