# Corrigendum: UV-B Pre-treatment Alters Phenolics Response to *Monilinia fructicola* Infection in a Structure-Dependent Way in Peach Skin

**DOI:** 10.3389/fpls.2018.01747

**Published:** 2018-11-29

**Authors:** Marco Santin, Susanne Neugart, Antonella Castagna, Martina Barilari, Sabrina Sarrocco, Giovanni Vannacci, Monika Schreiner, Annamaria Ranieri

**Affiliations:** ^1^Department of Agriculture, Food and Environment, University of Pisa, Pisa, Italy; ^2^Department of Biological Sciences, Loyola University, New Orleans, LA, United States; ^3^Leibniz Institute of Vegetable and Ornamental Crops (IGZ), Großbeeren, Germany; ^4^Interdepartmental Research Center Nutrafood “Nutraceuticals and Food for Health, ” University of Pisa, Pisa, Italy

**Keywords:** flavonol glycosides, ultraviolet radiation, fruit, *Prunus persica*, post-harvest, brown rot

In the original article, there was an error. We erroneously reported that peach fruits were delivered to the labs located at the Department of Applied Genetics and Cell Biology at the University of Natural Resources and Life Sciences, Vienna (Austria). Instead, peaches were brought to the Department of Agriculture, Food and Environment (DAFE), University of Pisa, Pisa (Italy), where UV-B irradiation and fruit infection were carried out.

A correction has been made to Materials and Methods, Plant Material and UV-B Treatments, paragraph 1. Please find below the complete corrected sentence:

Organic peaches (*Prunus persica* cv. Royal Majestic, melting phenotype) were purchased from a local supermarket and brought to the laboratory of the Department of Agriculture, Food and Environment (DAFE), University of Pisa, Pisa (Italy).

The authors apologize for this error and state that this does not change the scientific conclusions of the article in any way. The original article has been updated.

## Conflict of interest statement

The authors declare that the research was conducted in the absence of any commercial or financial relationships that could be construed as a potential conflict of interest.

